# Detection of Earthquake-Induced Damage in a Framed Structure Using a Finite Element Model Updating Procedure

**DOI:** 10.1155/2014/410539

**Published:** 2014-01-16

**Authors:** Eunjong Yu, Seung-Nam Kim, Taewon Park, Sang-Hyun Lee

**Affiliations:** ^1^Department of Architectural Engineering, Hanyang University 222, Wangsimni-ro, Seongdong-gu, Seoul 133-791, Republic of Korea; ^2^Department of Architectural Engineering, Dankook University, Jukjeon-dong, Yongin-si 448-701, Republic of Korea

## Abstract

Damage of a 5-story framed structure was identified from two types of measured data, which are frequency response functions (FRF) and natural frequencies, using a finite element (FE) model updating procedure. In this study, a procedure to determine the appropriate weightings for different groups of observations was proposed. In addition, a modified frame element which included rotational springs was used to construct the FE model for updating to represent concentrated damage at the member ends (a formulation for plastic hinges in framed structures subjected to strong earthquakes). The results of the model updating and subsequent damage detection when the rotational springs (RS model) were used were compared with those obtained using the conventional frame elements (FS model). Comparisons indicated that the RS model gave more accurate results than the FS model. That is, the errors in the natural frequencies of the updated models were smaller, and the identified damage showed clearer distinctions between damaged and undamaged members and was more consistent with observed damage.

## 1. Introduction

Monitoring integrity of infrastructures without interruption of its function is the primary motivation in developing vibration-based damage detection methods. This method relies on the fact that changes in structural properties, such as damage, affect the overall dynamic properties of the structure. The general approach to detecting damage in this method is to establish analytical models which represent the reference state and damaged state of the structure and then to investigate the change in structural properties between the two analytical models. Thus, a relevant parameter identification method yielding the optimal analytical model which represents the observed behaviors of a structure is essential. Several approaches have been explored to solve this inverse problem and are summarized by Doebling et al. [1, 2], Farrar et al. [3], Humar et al. [4], and Brownjohn [5]. The numerical optimization procedures that can be used in the field of damage detection are divided into two categories. One is based on pattern recognition algorithms such as artificial neural networks, which find an optimal parameter set from a relationship previously established using numerous parameter-response pairs [1, 6]. The other is the model updating approach which iteratively adjusts parameters (the stiffness, mass, and/or damping parameters) of the FE model to minimize differences in measured dynamic properties and those of the FE model [7]. Damage of the structure is evaluated by comparing the updated models of the reference state and damaged state.

In the FE model updating method, structural properties that represent the stiffness of the members in the analytical model, such as the modulus of elasticity or sectional property, are usually selected as the updating parameters. At this point, to obtain accurate results, it is important to establish a relevant analytical model and choose appropriate updating parameters so that the effects of damage on the dynamic properties can be accurately replicated. For example, if the flexural stiffness values of members were chosen as the updating parameters when the analytical model was not sufficiently discretized, it would be impossible to replicate the dynamic properties of a framed structure that has concentrated damage at only a few joints or portions of a few beam-column members. A typical problem arising from the use of an irrelevant model is that the identified damage tends to diffuse or spread out into other members [8], which makes parameter identification and subsequent damage identification inaccurate and difficult.

Another issue for accurate damage detection is improvement of accuracy in parameter identification from measured data. Generally, including many kinds of data as observation is advantageous since more information can be supplied and the ill-posedness of the inverse problem can be mitigated. The modal data (the natural frequencies and mode shapes obtained from various system identification methodologies) are frequently used to represent the dynamic properties of the structure. Even though parameter identification based on the model data is simple, replication of behavior in higher modes is impossible because it is generally difficult to identify the modal properties of higher modes. Thus, it is beneficial to include the frequency response function (FRF) to the modal properties as an observation for parameter identification to provide more information. When more than two kinds of measured data are used, the relative contribution of each type should be properly adjusted because the amounts of data and noise in each are different. The weighting factors are used for this purpose. Previous literature indicates that the weighting factor should be adjusted based on the variance of noise contained in each type of measured data [9, 10]. However, in reality, such information is not generally provided; thus, the weighting factors are determined by engineering judgment or on a trial-and-error basis. Therefore, results may differ depending on the choice of weighting factors, which makes parameter identification uncertain and inaccurate.

In this study, a model-updating-based damage detection procedure for framed structures subjected to seismic damage was proposed. For efficient detection of concentrated damage at the ends of a beam-column member, which is a typical damage pattern in framed structures subjected to major earthquakes, a modified frame element which includes the rotational springs at its ends was used in the analytical model. Both the FRF and natural frequencies were used as measured data for the FE model updating, and a procedure to obtain appropriate weighting factors is presented. The proposed procedure was applied to damage detection in a five-story one-bay reinforced concrete test structure subjected to earthquake damage. The accuracy of damage detection using the proposed procedure was compared with that of the conventional approach which uses conventional frame elements for the FE model and the flexural stiffness of the individual frame members as the updating parameters.

## 2. Frame Elements with Rotational Spring 

The basic premise of seismic design for framed structures is to ensure ductile behaviors even though some parts of the structures may behave nonlinearly. Under large earthquake excitations, typical damage in moment-resisting frames starts with the plastic hinge formulation (a concentration of flexural yielding) at the ends of the frame members. To properly replicate the nonlinear behavior of framed structures subjected to seismic damage, modified frame elements which include rotational springs at both ends were adopted in the analytical model which will be used in the FE model updating procedure.

**Figure 1:**
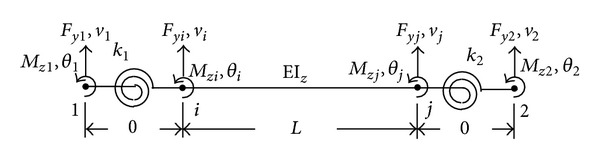
Frame element with rotational springs.

The stiffness matrix of a frame element with rotational strings at both ends can be derived (1) through the static condensation of the three-element model (a conventional frame element and two zero-length rotational springs at both ends) as shown in Figure 1 [11, 12]. Consider

(1) K=αEIzL[12L2(1+α1+α2α1α2)6L(1+2α2)−12L2(1+α1+α2α1α2)−6L(1+2α1)4(1+3α2)6L(1+2α2)2—12L2(1+α1+α2α1α2)6L(1+2α1)Sym.—4(1+3α1)],

where *α* = *α*_1_ *α*_2_/(*α*_1_ *α*_2_ + 4*α*_1_ + 4*α*_2_ + 12), and *α*_1_ and *α*_2_ are the stiffness indices of the rotational springs at the left and right ends of the frame element, respectively. The stiffness index is defined as the ratio of the stiffness of the rotational spring to the flexural stiffness of the frame element. The value of the stiffness index is zero for a pin connection and infinity for a rigid connection. If the stiffness indices were directly incorporated into the FE model updating procedure as the updating parameter, their values would have a range of zero to infinity. Since it is difficult to obtain the infinity in numerical calculations, the stiffness index is converted into a connection percentage [12] as

(2) ri=αi3+αi.

In (2), because the range of the connection percentage is 0 ≤ *r*_*i*_ ≤ *l*, the numerical implementation becomes much easier. The stiffness matrix using the connection percentage instead of the stiffness index is shown in (3). Thus, for updating framed structures which have concentrated damage at the member ends, the values for *r*_1_ and *r*_2_ are sought using the numerical updating procedure. Consider

(3) K=EIzL[12L2(r1+r2+r1r24−r1r2  )6L(r1r2+2r14−r1r2)4(3r14−r1r2)−12L2(r1+r2+r1r24−r1r2)−6L(r1r2+2r24−r1r2)6L(r1r2+2r14−r1r2)2Sym.12L2(r1+r2+r1r24−r1r2)6L(r1r2+2r24−r1r2)4(3r24−r1r2)].

Analytical models for framed structures are generally established using conventional frame elements (thus, the stiffness matrix for a single element does not contain the parentheses at each coefficient in (3)), and the model updating procedure may be applied to replicate measured dynamic behavior by adjusting the flexural stiffness (EI_*z*_). However, in this case, the behavior of a frame structure with plastic hinges cannot be accurately replicated. That is because the stiffness matrix with arbitrary *α*_1_  and *α*_2_ in (1) cannot be obtained by adjusting the flexural stiffness (EI_*z*_) in the stiffness matrix for conventional frame members. To obtain more accurate results, finer element discretization will be needed, which requires a large number of updating parameters and a substantial increase in computation cost.

## 3. FE Model Updating Procedure 

### 3.1. Overview of FE Model Updating Procedure

The FE model updating procedure used in this study is based on a nonlinear least-squares method which minimizes the difference between the measured and analytical frequency response functions and the natural frequencies using the sensitivity matrix and residual vector appended by the side constraints [13, 14]. The procedure was reorganized for acceleration measurement and base excitation and has been summarized.

The equation of motions of a system with *n* degrees-of-freedom subjected to base accelerations ${\ddot{u}}_{g}$ is expressed as

(4) Mu¨(t)+Cu˙(t)+Ku(t)=−MLu¨g(t),

where **M**, **C**, and **K** are (*n* × *n*) mass, damping, and stiffness matrices of the system, respectively, and **L** is the influence vector indicating the location of the DOFs being excited by ${\ddot{u}}_{g}(t)$. Equation (4) can be transformed into the frequency domain using the dynamic stiffness matrix **B**(*ω*) = **M** ^−1^(−**M** *ω* ^2^ + *i* **C** *ω* + **K**) and the transfer function vector $H\left(\omega \right)=u(\omega )/{\ddot{u}}_{g}(\omega )$. Consider

(5) B(ω)H(ω)=−L.

For updating, the transfer function **H**(*ω*) is replaced with $\tilde{H}\left(\omega \right)$ which is obtained from the measured base acceleration ${\tilde{\ddot{u}}}_{g}(\omega )$ and absolute story acceleration ${\tilde{\ddot{u}}}_{t}(\omega )$ as follows:

(6) H~(ω)=−1ω2(u¨~t(ω)u¨~g(ω)−L).

For model updating, the dynamic stiffness matrix **B**(*ω*) is replaced by **B**(**p**, *ω*), which is a function of a set of updating parameter vectors, *p* = [*p*_1_,*p*_2_,…,*p*_*m*_]^*T*^. Any geometric or material properties that were used in constructing the structural matrices **M**, **C**, and **K** can be used as the updating parameters. For the present problem, parameters affecting the stiffness of the member, such as the flexural stiffness EI_*z*_ or the connection percentage, were chosen as the updating parameters. An optimal parameter vector is one that minimizes the Euclidean norm of the error vector in

(7) ||εF(p)||2=||L+B(p,ω)H~(ω)||2.

In the nonlinear least-squares solution scheme, **B**(**p**, *ω*) is linearized using a Taylor series expansion truncated at the first order as

(8) B(p,ω)≈B(pk,ω)+∑i=1m∂B(pk,ω)∂piΔpi.

In addition, setting the norm of the error vector in (7) to zero, we get

(9) ∑i=1m∂B(pk,ω)∂piH~(ω)Δpi=L+B(pk,ω)H~(ω).

The gradient of *B*(*p*, *ω*) in the right side is defined at *p*_*k*_ (parameter vector at *k*th iteration) over the frequency range *ω* = [*ω*_1_, *ω*_1_, …, *ω*_*s*_] with respect to the *i*th parameter. Equation (9) can be rewritten as an (*s* × *m*) sensitivity matrix *S*_*k*_^*F*^(*p*_*k*_, *ω*) and an (*s* × 1) residual vector *r*_*k*_^*F*^(*p*_*k*_, *ω*) as

(10) SkF(pk,ω)Δpk=rkF(pk,ω).

The sensitivity matrix based on the natural frequency can be obtained in a similar manner. The norm of the error vector in the analytical natural frequencies ***λ***(*p*) and measured frequencies $\tilde{\lambda }$ can be expressed as

(11) ||εN(p)||2=||λ(p)−λ~||2.

Similarly, using a Taylor series expansion truncated at the first order, the least-squares equation is obtained as

(12) ∑i=1m∂λ(pk)∂piΔpi=λ(pk)−λ~.

Similar to (10), the updating equation with respect to the natural frequency can be written using the sensitivity matrix **S**_*k*_^*N*^(**p**_*k*_) and the residual vector **r**_*k*_^*N*^(**p**_*k*_) as

(13) SkN(pk)Δpk=rkN(pk).

Combining the equations for the FRF and the natural frequency in (10) and (13) yields

(14) Sk(pk,ω)Δpk=rk(pk,ω),

where

(15) Sk(pk,ω)=[SkNRe(SkF)Im(SkF)],  rk(pk,ω)=[rkNRe(rkF)Im(rkF)].

The solution can be found using an iterative calculation until the incremental solution Δ*p*_*k*_ or the residual vector **r**_*k*_(**p**_*k*_, *ω*) diminishes sufficiently (below the convergence criteria),

(16) pk+1=pk+Δpk.

### 3.2. Weighting Factor

Equation (14) is a combination of FRF-based and natural-frequency-based equations. The relative influence of each type of data on the solution can be controlled using the weighting factor as

(17) ST(p,ω)WS(p,ω)Δp=ST(p,ω)Wr(p,ω),

where **W** is a diagonal matrix containing the weighting factors. The subscript *k* was omitted for brevity.

Three aspects must be considered in determining the weighting factors: (a) even though groups of data may be measured in different units, weighted quantities should be in the same units; (b) relatively accurate measurements are weighted more heavily than inaccurate measurements; and (c) the numbers of observations in each group should be considered to ensure the desired contributions.

Typically, the size of the sensitivity matrix based on the FRF is much larger than one based on natural frequencies. Thus, a larger weighing factor should be used for the natural frequencies to ensure an equivalent contribution. Otherwise, the natural frequencies will have very little impact on the solution. From statistical theory, it is known that the best weighting matrix for the least-squares problem is the inverse of the covariance matrix of the measurement uncertainty [9, 10]. However, since this information is not available in most cases, the weighting factors are usually chosen by engineering judgment by considering the presumed importance of each measurement and the amount of noise it contains. Therefore, when different values for the weighting factors are chosen, the solution differs, which makes the model updating uncertain.

In this study, the weighting factors were determined by considering the three aspects mentioned above. The objective function for the weighted least-squares problem in (17) can be alternatively expressed as

(18) J(Δp)=min(||εFa||2+χ||εNa||2)=min(∑i=1s1s[(εF)i]2+χ∑i=1mwim[(εN)i]2),

where *s* and *m*  indicate the numbers of observations for the FRF and the natural frequency, respectively. Thus, the error norms from each type of measurement were divided by the number of observations for comparable contributions from both observation groups. The value *w*_*i*_ is the reciprocal of the measured natural frequency vector as in (19) and is applied to squared error norm of the natural frequency to equalize the units with

(19) wi=[1λ1~,1λ2~,…,1λm~  ].

The value *χ* is the scaling factor that controls the relative contribution of the natural frequency group. Since information regarding the accuracy of the observations (i.e., the covariance matrix of the measurement error) was not provided in this problem, the values of *χ* are determined so that the sum of the average FRF norm and the natural frequency norm can be minimized. That is, the parameters are estimated by quantifying the fit between the FRF and the natural frequency values for the value of *χ* which minimizes

(20) min(||εFa||+||εNa||).

**Figure 2:**
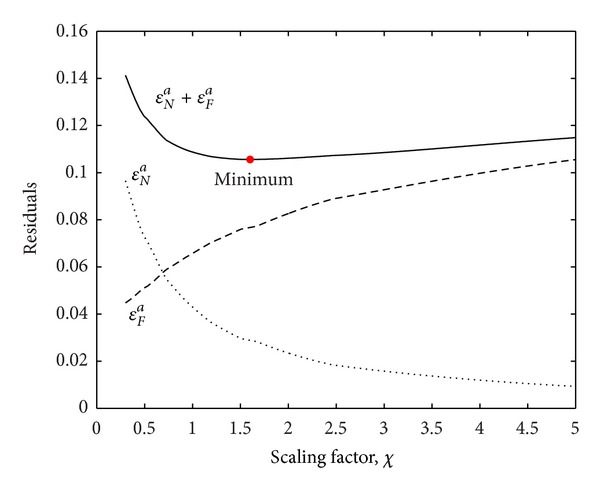
Change in average error norm with scaling factor *χ*.

The components of the diagonal matrix *W* can be derived from the factors in (18) and *χ* satisfying (20).  Figure 2 is an example showing the variation of the average norms of the FRF and natural frequency errors, respectively, with variation of the scaling factor. Since *χ* represents the relative contribution of the natural frequency to the FRF, it is shown that the average error norm of the natural frequency decreased with increasing *χ*.

### 3.3. Grouping of Parameters

The parameter identification problem is generally an ill-conditioned problem. Typically, in the solution of an ill-conditioned problem using the least-squares equation, a few parameters exhibit large changes from their initial values. Ill-conditioning occurs when two or more parameters have very similar effects on the response of the system and the measurements are contaminated by noise. The regularization method [15] and grouping of parameters [16] are numerical techniques commonly used to alleviate this phenomenon. In this paper, grouping of the updating parameters and appending side constraints were used to limit the variation of similar parameters based on the cosine angle of the sensitivity vectors of each parameter [17]

(21) |pi−pj|<1−angle(Si,Sj),   if  angle(Si,Sj)>clim,

where *S*_*i*_ and *S*_*j*_ are the sensitivity vectors (the *i*th and *j*th columns of the sensitivity matrix *S*) with respect to the updating parameters *p*_*i*_ and *p*_*j*_, respectively. The term angle (*S*_*i*_, *S*_*j*_) denotes the cosine angle between the vectors *S*_*i*_ and *S*_*j*_, as given by

(22) angle(Si,Sj)={SiT·Sj}{SiT·Si}{SjT·Sj}.

Equation (21) represents the constraints placed on the relative variations between any two parameters. If two parameters have similar effects on the response (e.g., the angle between the two sensitivity vectors is larger than a given limit *c*_lim_), then the difference between the two updated parameters is restricted to remain within a range that depends on their degree of similarity. This requirement forces the updating parameters to change similarly if they have similar effects on the structural response. This method has the effect of reducing the actual number of parameters while keeping a large set of parameters. The value for *c*_lim_ should be assigned so that the number of independent variables in the system is equal to the number of responses (channels).

## 4. Test Structure 

**Figure 3:**
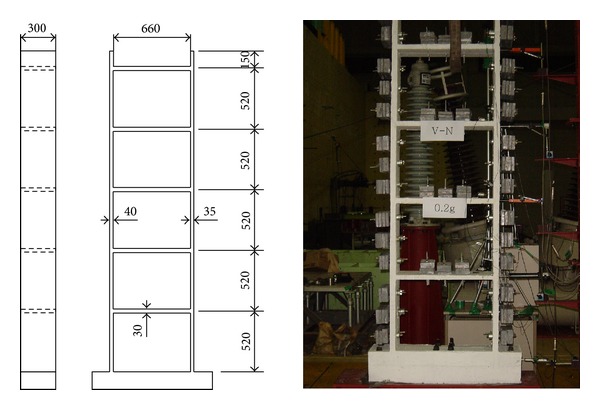
Test structure.

The proposed FE model and model updating procedure were applied to identify seismic damage in a test structure using the acceleration data measured during the shaking table test. The test structure was a one-bay, five-story, reinforced-concrete wall-slab building.  Figure 3 shows the test structure and its dimensions. The dimensions of the members were scaled down to 1/5 of the typical member size in actual wall-slab-type buildings. The diameters of deformed bars and the coarse aggregate in the reinforced concrete were reduced by the geometric scale factor. In addition, mass blocks were attached to the walls and the slabs to adjust the natural frequencies to $1/\sqrt{5}$ of the prototype structure to maintain an identical stress and strain relationship between the prototype structure and the scaled model. The shaking table tests were performed in the out-of-plane direction from the walls on a uniaxial earthquake simulator. The test structure was subjected to the El Centro ground history scaled to six different PGA levels of 0.06 g, 0.12 g, 0.20 g, 0.30 g, 0.40 g, and 0.5 g in increasing order. Accelerations at the base and on each floor of the test structure were measured.

**Figure 4:**
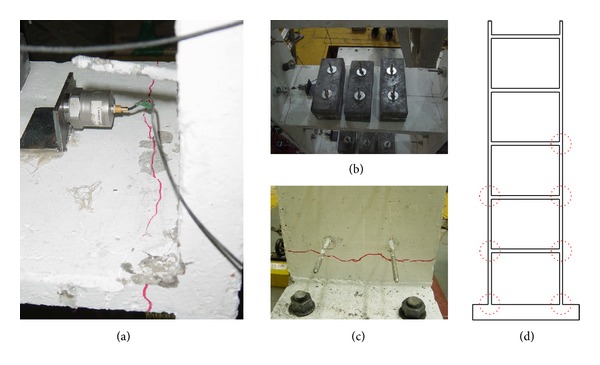
Crack patterns observed after each stage of the tests: (a) the right end of the third-floor slab after 0.20 g shaking, (b) the third- and second-floor slabs after 0.30 g shaking, (c) the base of the first-story wall after 0.50 g shaking, and (d) locations of observed cracks.

After each stage of the test, cracks in the structure were inspected and recorded. With increased ground acceleration amplitude, some members of the test structure suffered significant damage from concentrated cracks at the ends of the members.  Figure 4 shows the damage of the test structure observed at selected stages of the test. No noticeable cracks were observed at 0.06 g and 0.12 g shaking. At 0.20 g shaking, the first major crack occurred at the right end of the third-floor slab and minor cracks were observed at the base of the first-story wall on the right side. After the 0.30 g shaking, cracks were observed at the left side of the third-floor slab, both ends of the second-floor slab, and the right end of the fourth-floor slab. At 0.40 g shaking, no new major cracks were observed; however, the existing cracks, especially at the slab ends, were significantly enlarged. Finally, at 0.5 g shaking, the crack at the base of the first-story wall on the left side was enlarged.

The natural frequencies and modal damping ratios of the test structure at each stage of shaking were identified from the measured acceleration data using N4SID (subspace state-space system identification), which is a time domain system identification method [17].  Table 1 summarizes the identified natural frequencies and modal damping ratios for each stage of shaking.

As indicated in the table, modal properties up to the fourth mode could be identified. With increased vibration amplitude, the fundamental natural frequency gradually decreased and finally reached about 30% of that of the 0.06 g shaking. The damping ratio of the fundamental mode of the 0.50 g shaking was increased by about eight times that of the initial test, which is believed to be the result of hysteretic damping due to the nonlinear behavior of the test structure. The table shows that the decrease in the natural frequency at the 0.30 g shaking was as large as 40% compared with the previous step, which indicates that the change in the overall stiffness distribution occurred somewhat abruptly during the 0.30 g shaking.

## 5. Damage Identification of the Test Structure

### 5.1. Model Updating for Reference (Undamaged) Model

The initial model used to determine the reference model was composed based on the information obtained from the material test results and the measured dimensions of the members. Young's modulus for concrete was evaluated with the compressive strengths from core sample tests using the equation in ACI

(23) Ec=4700fc′.

The average Young modulus of concrete was found to be 24.77 GPa. The stiffness of the members of the initial model was determined based on the moment of inertia of a gross (uncracked) section. The story mass was obtained from the self-weight of the members and the mass blocks attached to the test structure having values of 108.87 kg for the roof floor and 138.08 kg for the other floors. The resulting natural frequencies of the initial FE model were 3.63, 11.92, 22.76, 35.89, and 47.74 Hz for the first to fifth modes. The basic assumptions used in the initial model were that (i) the mass of the structure was concentrated at floor level, (ii) the floor was rigid in in-plane, and (iii) the damping properties of the structure were those of classical damping.

The dynamic properties (FRF and identified natural frequencies) at 0.06 g shaking were used to determine the reference FE model. In the model updating for the reference FE model, the connection percentages of all rotational springs were assumed to be one, which represents a rigid connection. These values were not selected as updating parameters. Both of the flexural stiffness and connection percentage cannot be used as updating parameters at the same time because selecting many parameters, some having similar influences on the dynamic behavior of the structure, is a source of ill-conditioning. For the undamaged state, an assumption of rigid connections was appropriate because no cracks were observed at this stage.

**Figure 5:**
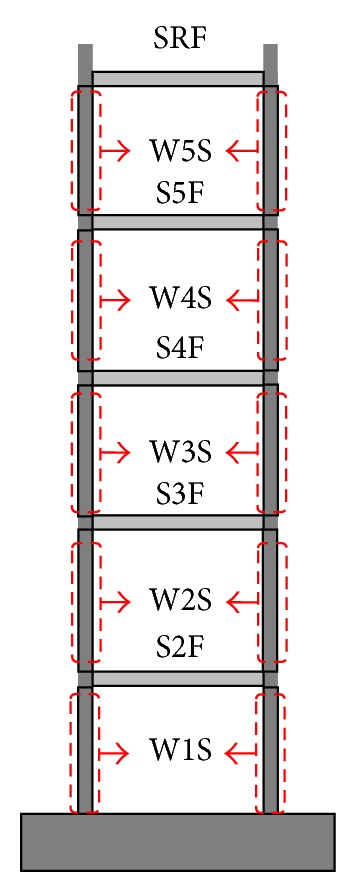
Updating parameters for initial analytical model.

The total of ten updating parameters consisted of five for the columns and five for the slabs and was used as shown in Figure 5. The stiffness of two walls in a story was grouped together because the contribution of each wall on the global behavior of the structure should be almost identical. Tables 2 and 3 show the updated parameters and natural frequencies of the updated FE model. As can be seen in the tables, the stiffness of the members in the updated model was higher than the initial guess. This is because the flexural stiffness of the initial model was estimated based on the modulus of elasticity of concrete which was defined as the secant modulus of the stress-strain curve for concrete at 40% of the ultimate concrete strength. However, the maximum stress induced at the 0.06 g shaking was significantly below 0.4*f*_*c*_′.

### 5.2. Model Updating for Damaged Model and Damage Identification

After obtaining the reference model, which corresponds to the state of the structure after 0.06 g shaking, model updating for the rest of the shaking stages was performed, and the damage at each stage was evaluated using the updated parameters. However, the connection percentages of the rotational springs, instead of the effective flexural stiffness EI_*z*_, were chosen as the updating parameters for the damaged model. The flexural stiffness of the frame members was fixed to the values obtained from the model updating for the undamaged structure. This is to reflect the typical damage pattern of framed structures subjected to earthquake excitations, in which the damage of the structure is concentrated at the member ends through the formulation of plastic hinges. Thus, it was expected that degradation of stiffness due to the plastic hinge formulation would be captured by the stiffness change of the rotational springs.

**Figure 6:**
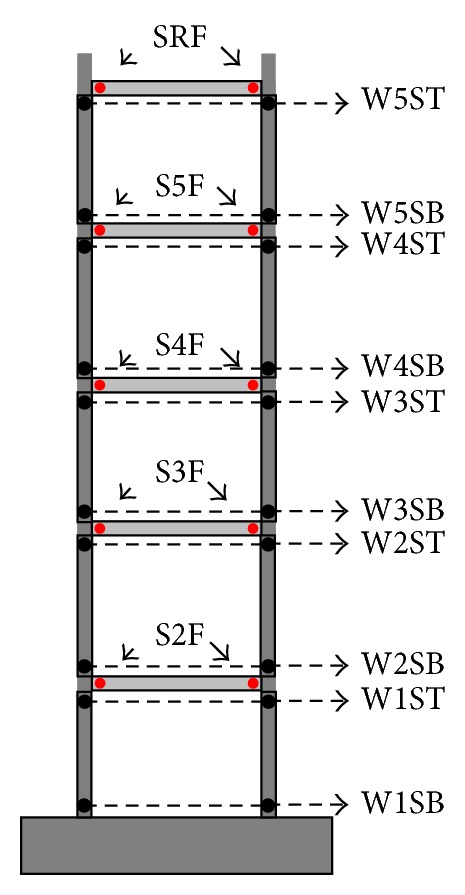
Updating parameters for damaged model.

The number of rotational springs used in the damaged model was 30, which is twice the number of frame members. The number of parameters was reduced using a similar grouping scheme. The influence of the spring located at the bottom of one wall is almost identical to the spring located at the bottom of the other wall. The two bottom springs of a story were grouped together. Likewise, the two top springs of a story and the two springs located at both ends of a beam were grouped together. Therefore, a total of 15 parameters, three parameters per story, were used for the damaged model.  Figure 6 shows the updating parameters for each story. In addition, another model updating based on the conventional FE model, which uses the flexural stiffness of the frame members as the updating parameters, was performed separately and both sets of results were compared. In the model updating based on flexural stiffness (FS model), the number of updating parameters was 10, as in the undamaged model. In both cases, the updated parameters from the previous stage were used as the initial values for the current shaking stage.

Table 4 shows the analytical natural frequencies of the updated models at each stage when the stiffness of the rotational springs (RS model) and flexural stiffness (FS model) were used as updating parameters. The table also shows the comparison with the measured (identified) natural frequencies. The percentage error in the table indicates the deviation of the analytical frequencies from the measured ones. As can be seen in the table, the errors in the RS model were much lower than in the FS model, which implies that the RS model is capable of representing the behavior of the test structure more accurately than the FS model. In both cases, the error increased as the intensity of excitation increased. This is probably because the test structure behaved nonlinearly during large amplitude excitations, although parameter identification was made based on a linear model.

**Figure 7:**
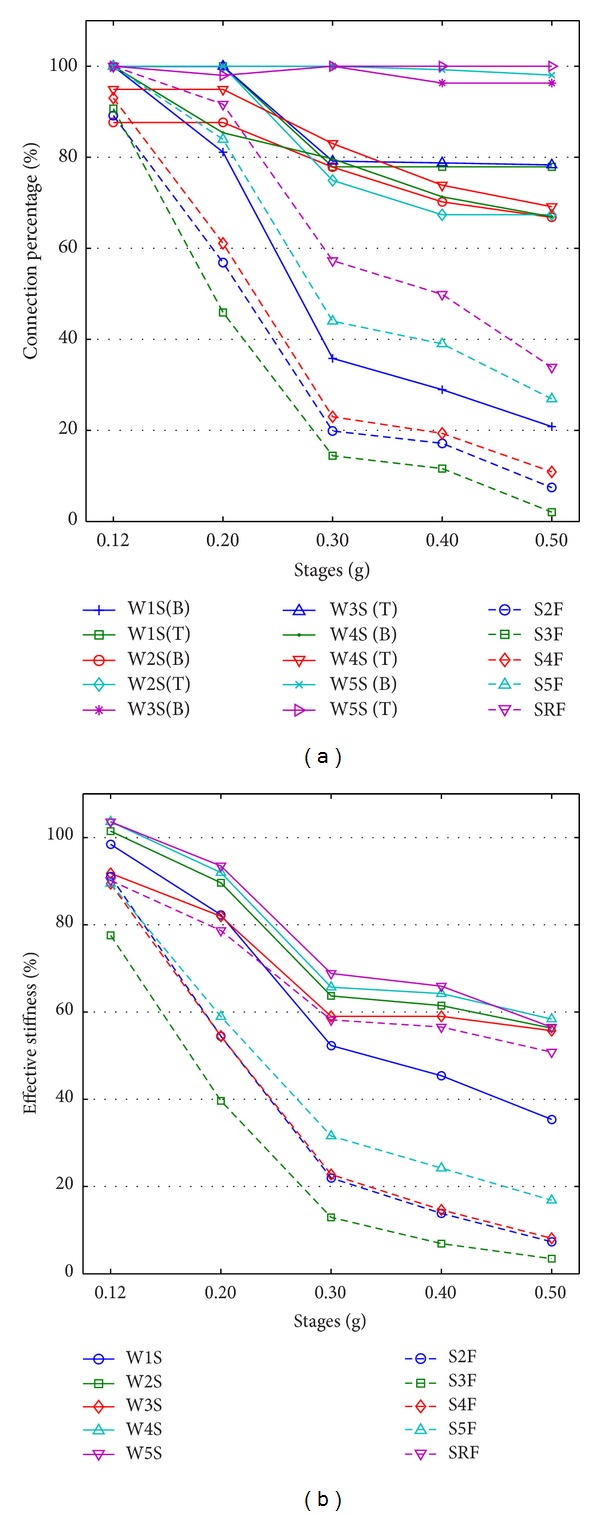
Change in stiffness ratios: (a) FS model, and (b) RS model.

Table 5 summarizes the updated parameters at each stage of shaking and is represented as graphs in Figure 7. The values in the table and graphs were normalized to the values of the undamaged model. Damage of the structure can be inferred from the changes of the parameters. As indicated in Figure 5, major cracks were observed at both ends of the second- and third- floor slabs, the right end of the fourth slab, and the bottom of the first-story wall. In both the FS and RS models, the parameter set that showed an apparent stiffness drop included the observed damaged members and some of the undamaged members. The overall distribution in stiffness ratios appeared to be closer to visual inspection in the case of RS model. In the FS model, the stiffness degradation of the slab on the fifth floor was relatively significant, although there was no major crack observed. In addition, the stiffness ratio of the wall at the first story was not distinctively low compared to the other wall members. At 0.50 g shaking, the stiffness of the walls on the second through fifth stories decreased by about 40%, although there was no major crack observed.

The distribution of stiffness ratios for RS model enabled a clear distinction between damaged and undamaged members. The range of stiffness ratios increased (0.98 to 0.02 in the case of 0.50 g shaking). The stiffness ratios of the rotational springs of the walls indicated that the stiffness drop at the bottom of the first-story wall was most significant, while the stiffness ratios of the other rotational springs did not decrease considerably.

As mentioned previously, the stiffness of a frame member with nonuniform damage along its length cannot be simulated accurately by adjusting the flexural stiffness EI_*z*_ alone. The stiffness degradation, including undamaged members in the FS model, can be seen as a consequence of “diffusion” or “spreading out” of damage in which the stiffness of the undamaged members was affected by the damage in other members. On the other hand, in the RS model, this diffusion effect was greatly reduced. The ability of the FE model to replicate the global behavior of a structure with the progress of damage is important for accurate damage detection. The RS model is effective in detection of earthquake-induced damage in framed structures.

## 6. Conclusions

In this paper, damage of a 5-story framed structure induced by shaking table testing was identified and quantified using an FE model updating procedure. The model updating method in this study used the frequency response functions and natural frequencies as observations, which were obtained from acceleration measurements during the test. Generally, it is known that using many kinds of observations is advantageous, since more information can be supplied and the ill-posedness of the inverse problem can be mitigated. However, for more accurate results, appropriate weighting and scaling between the groups of observations are needed. In this study, a procedure to determine the rational weightings of different groups of observations was proposed and applied. In addition, considering the typical features of earthquake-induced damage in framed structures, an FE model for updating was constructed using a modified frame element which includes rotational springs at both ends, and the rotational springs were chosen as updating parameters. When conventional frame elements were used and the flexural stiffness of individual members was chosen as updating parameters, the effects of concentrated damage at the members' ends were not replicated with sufficient accuracy. Therefore, damage detection based on this type of model updating may contain large inaccuracy. In this paper, model updating using conventional frame elements (FS model) and modified frame elements (RS model) was conducted separately. Then, the degrees of damage evaluated using the results of the two model updating methods were compared. The comparison showed that the errors in the natural frequencies of the updated FE models at 0.50 g shaking were about 8–23% in the FS model and 6–17% in the RS model, which indicated that the RS model is capable of representing the dynamic behavior of the structure more accurately. The location and severity of damage were identified from changes in the updated parameter values in each case. The damage identified by the FS model showed somewhat diffused; that is, the stiffness ratios of severely damaged members were not reduced sufficiently, and some undamaged members showed large stiffness drops although no major cracks were observed. On the other hand, the damage identified by the RS model showed clear distinctions between damaged and undamaged members, and were more consistent with observed damage.

## Figures and Tables

**Table 1:** Natural frequencies and damping ratios of the test structure.

	Natural frequencies (Hz)				Damping ratios (%)			
	1st	2nd	3rd	4th	1st	2nd	3rd	4th
El Centro 0.06 g	4.01	13.08	25.15	41.60	2.56	2.78	3.87	5.68
El Centro 0.12 g	3.78	12.65	24.85	40.80	3.60	3.42	3.88	6.51
El Centro 0.20 g	3.00	11.06	22.42	39.73	10.43	5.19	7.20	11.16
El Centro 0.30 g	1.90	8.49	19.64	34.67	16.00	7.09	4.89	10.17
El Centro 0.40 g	1.59	8.16	18.55	33.40	15.90	5.72	5.30	10.83
El Centro 0.50 g	1.24	7.57	17.49	32.32	30.99	7.80	5.59	9.65

**Table 2:** Updating parameters for reference model.

Parameters	Value	Parameters	Value
Wall 1S	1.30	Slab 2F	1.14
Wall 2S	1.39	Slab 3F	1.31
Wall 3S	1.19	Slab 4F	1.16
Wall 4S	1.34	Slab 5F	1.03
Wall 5S	1.32	Slab RF	1.19

**Table 3:** Natural frequencies of reference model.

		1st	2nd	3rd	4th
Measured (0.06 g shaking)	Freq. (Hz)	4.01	13.08	25.15	41.60
Initial model	Freq. (Hz)	3.63	11.92	22.76	35.89
	Percent error	−11%	−9%	−11%	−15%
Updated model	Freq. (Hz)	4.01	13.03	25.38	40.92
	Percent error	0%	0%	1%	−1%

**Table 4:** Comparison of measured and analytical natural frequencies.

Stage	Mode	Measured Freq. (Hz)	RS model		FS model	
			Freq. (Hz)	Percent error	Freq. (Hz)	Percent error
PGA 0.12 g	1st	3.78	3.78	0.0%	3.78	0.0%
	2nd	12.65	12.73	0.6%	12.68	0.2%
	3rd	24.85	24.82	−0.1%	24.99	0.6%
	4th	40.80	40.60	−0.5%	40.13	−1.6%

PGA 0.20 g	1st	3.00	2.90	−3.3%	3.13	4.3%
	2nd	11.06	11.02	−0.4%	11.17	1.0%
	3rd	22.42	22.61	0.8%	22.14	−1.2%
	4th	39.73	37.98	−4.4%	36.00	−9.4%

PGA 0.30 g	1st	1.90	1.83	−3.7%	2.03	6.8%
	2nd	8.49	7.84	−7.7%	8.79	3.5%
	3rd	19.64	17.73	−9.7%	17.80	−9.4%
	4th	34.67	31.67	−8.7%	29.38	−15.3%

PGA 0.40 g	1st	1.59	1.68	5.7%	1.82	14.5%
	2nd	8.16	7.27	−10.9%	8.04	−1.5%
	3rd	18.55	16.80	−9.4%	16.10	−13.2%
	4th	33.40	30.55	−8.5%	26.45	−20.8%

PGA 0.50 g	1st	1.24	1.32	6.5%	1.45	16.9%
	2nd	7.57	6.31	−16.6%	6.98	−7.8%
	3rd	17.49	15.65	−10.5%	14.90	−14.8%
	4th	32.32	29.41	−9.0%	24.78	−23.3%

**Table 5:** Updated parameters normalized with those of undamaged model.

		0.12 g		0.20 g		0.30 g		0.40 g		0.50 g	
		RS	FS	RS	FS	RS	FS	RS	FS	RS	FS
Wall 1S	Bot.	1.00	0.99	0.81	0.82	0.36	0.52	0.29	0.45	0.21	0.35
	Top	1.00		1.00		0.79		0.79		0.78	

Wall 2S	Bot.	1.00	1.02	1.00	0.90	0.78	0.64	0.78	0.62	0.78	0.56
	Top	1.00		0.85		0.80		0.71		0.67	

Wall 3S	Bot.	0.88	0.92	0.88	0.82	0.78	0.59	0.70	0.59	0.67	0.56
	Top	0.95		0.95		0.83		0.74		0.69	

Wall 4S	Bot.	1.00	1.04	1.00	0.92	0.75	0.66	0.67	0.64	0.67	0.58
	Top	1.00		1.00		1.00		0.99		0.98	

Wall 5S	Bot.	1.00	1.04	1.00	0.94	1.00	0.69	0.96	0.66	0.96	0.57
	Top	1.00		0.98		1.00		1.00		1.00	

Slab 2F		0.89	0.91	0.57	0.55	0.20	0.22	0.17	0.14	0.07	0.07
Slab 3F		0.91	0.78	0.46	0.40	0.14	0.13	0.12	0.07	0.02	0.03
Slab 4F		0.93	0.89	0.61	0.55	0.23	0.23	0.19	0.15	0.11	0.08
Slab 5F		1.00	0.90	0.84	0.59	0.44	0.32	0.39	0.24	0.27	0.17
Slab RF		1.00	0.90	0.92	0.79	0.57	0.58	0.50	0.57	0.34	0.51
